# RNA biology takes root in plant systems

**DOI:** 10.1002/pld3.445

**Published:** 2022-09-06

**Authors:** David Yu, Lauren McKinley, Yachi Nien, Wil Prall, Allison Zvarick

**Affiliations:** ^1^ Department of Biology The Pennsylvania State University University Park PA USA; ^2^ Department of Chemistry The Pennsylvania State University University Park PA USA; ^3^ Department of Biology The University of Pennsylvania Philadelphia PA USA

**Keywords:** plant, RNA, transcriptional regulation, translational regulation

## Abstract

Advances in RNA biology such as RNAi, CRISPR, and the first mRNA vaccine represent the enormous potential of RNA research to address current problems. Additionally, plants are a diverse and undeniably essential resource for life threatened by climate change, loss of arable land, and pollution. Different aspects of RNA such as its processing, modification and structure are intertwined with plant development, physiology and stress response. This report details the findings of researchers around the world during the 23rd Penn State Symposium in Plant Biology with a focus in RNA biology.

## 1 INTRODUCTION

RNA has gained prominence due to its versatile nature—RNA can possess enzymatic activity, adopt multiple structural conformations with impacts on function, and sense environmental factors such as heat or pH as well as metabolites. The advent of the first mRNA vaccines for COVID‐19 shows how invaluable RNA research can be to providing solutions for modern problems. The 23rd Penn State Symposium in Plant Biology was held May 18–20, 2022, at the University Park campus of The Pennsylvania State University. It gathered RNA aficionados to share their exciting, new discoveries in plant RNA biology. This conference featured over 100 participants, and research spanning 20 countries, as well as 24 states within the United States.

In this meeting report, we describe research that was presented on RNA processing that occurs within the cell, such as alternative splicing, noncanonical RNA caps and RNA editing, RNA turnover, and RNA‐directed regulation of transcription and translation. We delve into new insights regarding short and long noncoding RNAs (ncRNAs) in epigenetic reprograming. Finally, we discuss the RNA‐based tool CRISPR, RNA sensing of environmental responses via RNA modification and structure, and RNA interactions outside the cell through its role in *trans*‐kingdom communication utilizing small RNAs (sRNAs).

### 1.1 RNA processing

The processing of pre‐mRNA into mature sequences controls localization and regulation of subsequent proteins, which in turn affects key processes such as plant development and stress response. Alternative splicing (AS) is a processing step that expands the transcriptome of eukaryotes through the formation of multiple isoforms of a gene, each capable of independent functions. This process has been shown to primarily occur cotranscriptionally in the spliceosome and through recruitment of additional RNA‐binding proteins (Reddy, 2007). Dr. Anireddy Reddy (Colorado State University, USA) demonstrated the significant impact different isoforms of serine/arginine rich RNA‐binding protein 45 (SR45), a known splicing regulator, can have on salt stress response in *Arabidopsis thaliana*. The two isoforms of SR45, SR45.1 (long) and SR45.2 (short), have been shown to function in different developmental processes including flowering and root growth, respectively (Zhang & Mount, 2009). Dr. Reddy utilized a *sr45* mutant of Arabidopsis, which is highly sensitive to salt stress, and isoform‐specific complementation by either *SR45.1* or *SR45.2*. The results showed that salt hypersensitivity in the *sr45* mutant was rescued by *SR45.1* but not *SR45.2*, perhaps due to regulation by *SR45.1* of Salt Overly Sensitive (SOS) or ABA‐related genes. This suggests the long isoform of SR45 is critical to regulate splicing in salt stress‐related pathways (Albaqami et al., 2019).

In addition to mRNA processing occurring cotranscriptionally, evidence presented by Dr. Artur Jarmolowski (Adam Mickiewicz University, Poland) suggested that pri‐miRNAs (the primary transcripts that give rise to microRNAs) are processed cotranscriptionally in Arabidopsis. Dr. Jarmolowski's work utilized “plant native elongating transcripts sequencing” (plaNET‐seq) to show that cotranscriptional processing of pri‐miRNAs occurs within Arabidopsis and that the process relies heavily on R‐loop formation at the transcriptional start site of the miRNA loci. Interestingly, growth conditions may impact whether a transcript is cotranscriptionally or posttranscriptionally processed, potentially due to regulation of R‐loop formation (Gonzalo et al., 2022).

Transcript diversity can also be regulated by RNA editing, albeit relatively rare in comparison with splicing. Examples of RNA editing include deamination (removal of an amine group from cytidine to give rise to uridine or C‐to‐U editing). C‐to‐U conversions are widespread in plant mitochondria and chloroplasts. Dr. Stephane Bentolila (Cornell University, USA) demonstrated the involvement of the RanBP2 zinc finger (RanBP2 Znf) domain in plant organelle RNA intron editing and splicing (Bentolila et al., 2021; Gipson et al., 2022). C‐to‐U editing of transcripts in plant organelles is carried out by small (<400 kD) protein complexes called editosomes. Dr. Bentolila's team identified a component of the editosome in chloroplasts, the Organelle Zinc finger 1 (OZ1) protein. OZ1 is required for C‐to‐U editing in chloroplasts. The only annotated domain in OZ1 is the RanBP2‐type zinc finger (Znf) domain. Mutation of key structural residues in the Znf domains showed that they are necessary for editing. Dr. Bentolila's team also investigated the function of OZ2, whose null mutation is embryo lethal. Genetic and biochemical analyses demonstrated that OZ2 is not an editing factor, but instead promotes the splicing of transcripts of several mitochondrial genes. These findings extend the known functional repertoire of the RanBP2 zinc finger domain in RNA splicing and editing in plant organelles.

### 1.2 RNA caps

Most plant mRNAs possess a canonical 5′ N7‐methylguanosine cap that is responsible for regulating stability, nuclear export, splicing, and translation initiation of the transcript (Ramanathan et al., 2016). However, several noncanonical caps derived from cellular metabolites, including the ATP analog nicotinamide adenine dinucleotide (NAD^+^), have been identified in plants and other eukaryotes (Wiedermannová et al., 2021). Dr. Xuemei Chen (University of California, Riverside, USA) presented a novel approach, strain‐promoted azide–alkyne cycloaddition (SPAAC) NAD‐seq, that her lab devised to identify NAD^+^ capped RNAs. Thousands of putative RNAs bearing an NAD^+^ cap were identified in Arabidopsis using NAD captureSeq (Wang et al., 2019). NAD captureSeq is a popular method employed to find NAD^+^ capped RNAs by enzymatically replacing the NAD^+^ cap with an alkynyl alcohol which is then biotinylated via copper‐mediated click chemistry (Cahová et al., 2015). Subsequently, the biotinylated RNA is pulled down using streptavidin followed by sequencing. There are several challenges with this method, though, including its reliance on copper, which is known to promote RNA degradation, and contamination by m^7^G capped transcripts (Hu et al., 2021). To combat these issues, SPAAC‐NAD‐seq instead utilizes a reactive azide, rather than an alkyne, to biotinylate NAD^+^ capped RNA through a SPAAC reaction, thereby eliminating the need for any copper in the reaction (Hu et al., 2021). Furthermore, Dr. Chen and coworkers added an immunodepletion step with an anti‐m^7^G antibody to remove contaminating transcripts with an m^7^G cap. To directly compare the two approaches, they performed both NAD captureSeq and SPAAC‐NAD‐seq on total RNA from Arabidopsis (Hu et al., 2021). While NAD captureSeq found 3,683 NAD capped RNAs, SPAAC‐NAD‐seq identified 5,642, and these were enriched in full length transcripts and those lower in abundance (Hu et al., 2021). Future work will focus on discovering enzymes that install and remove NAD^+^ caps and adapting SPAAC‐NAD‐seq to find RNAs with other noncanonical caps.

### 1.3 RNA turnover

Errors arise during RNA processing events, including inaccurate RNA splicing, which can create gene products that are deleterious to the organism. Quality control mechanisms exist to survey and remove aberrant mRNAs. Nonsense‐mediated mRNA decay (NMD) is a eukaryotic mRNA surveillance system that shapes the transcriptome by eliminating mRNAs that contain premature stop codon. Dr. Misato Ohtani (The University of Tokyo, Kashiwa, Japan) discovered that deficiency of NMD alters tissue‐specific cell redifferentiation. NMD‐deficient mutants formed adventitious roots instead of adventitious shoots, under the shoot induction condition of tissue culture (Chiam et al., 2019). mRNA half‐life analysis indicated shoot regeneration‐specific changes in mRNA stability for specific mRNA species, suggesting that selective mRNA degradation is crucial for stem cell specification during shoot regeneration. These findings suggest selective mRNA stability is key for stem cell conversion, especially from root type to shoot type.

mRNA abundance is controlled by the balance of two factors: its synthesis from transcription and its decay. mRNA turnover is also critical in cellular homeostasis. A major pathway in plants known as the cytoplasmic mRNA decay pathway consists of mRNA deadenylation followed by either 5′ to 3′ degradation via the decapping enzyme *VARICOSE* (VCS) and exonuclease digestion by *EXORIBONUCLEASE* (XRN4) or 3′ to 5′ degradation via *SUPPRESSOR OF VARICOSE* (SOV) or the exosome pathway (Sieburth & Vincent, 2018). Dr. Leslie Sieburth (University of Utah, USA) reported results using an Arabidopsis *trans*‐differentiation system, specifically the conversion of mesophyll cells into vascular cells, to interrogate the role of RNA decay (Sorenson et al., 2018). Changes in RNA half‐life quantified after application of the *trans*‐differentiation signal, a mixture of auxin, cytokinin, and bikinin, showed 9% shorter lived mRNAs, 13% longer lived mRNAs and 78% mRNAs without change. Dr. Sieburth reported only a small number of genes (~25) were solely regulated by decay which supports the idea of transcriptional balance between synthesis and decay. Dr. Sieburth also found a subset of *sov* mRNAs with shorter half‐lives, or high flux, but moderately increased abundance in comparison with WT. This indicates transcription plays a significant role in determining mRNA abundance. This phenomenon where significant changes in half‐life were measured but no significant changes in abundance were detected is an RNA decay defect feedback pathway known as RNA buffering. One key similarity for transcripts exhibiting RNA buffering is degradation involving mRNA decapping by VCS. These VCS‐dependent, high‐flux RNAs included genes known to function as environmental and developmental signals, which indicates probable regulatory function.

Dr. Pamela Green (University of Delaware, USA) presented her group's research on the elucidation of Arabidopsis *DCP1‐ASSOCIATED NYN ENDORIBONUCLEASE 1* (DNE1) targets. DNE1 is a cytoplasmic mRNA decay factor known to be involved in mRNA decapping and NMD (Chicois et al., 2018; Schiaffini et al., 2022). Analysis of the RNA degradome showed major targets of DNE1 are exons within the coding sequence and included a variety of targets such as uORFs, NMD‐sensitive transcripts, and, unexpectedly, NMD‐insensitive transcripts. DNE1 targets were also characterized by turnover rates twice as fast as nontargets. Furthermore, mutational analysis revealed all four aspartic acid residues in DNE1's NYN domain was required for endoribonuclease function. Using a *dne1* × *xrn4* mutant, Dr. Green observed an additive effect on decapping in comparison with *xrn4* which implicates DNE1 as a novel decapping effector. These reports provide evidence for DNE1's dual role as both an endoribonuclease and decapping interactor for a broad range of inherently unstable targets.

Ribosomes are an essential component of protein synthesis and account for most of the RNA within the cell. While ribosomal processes such as assembly and regulation have been elucidated in great detail, ribosome turnover is relatively unstudied. Dr. Gustavo MacIntosh (Iowa State University, USA) presented his work on the vacuolar RNA salvage pathway through *RNS2*, the major RNase in Arabidopsis, *autophagy‐related* (*ATG*) genes, and the RNA helicase SKI2. Dr. MacIntosh's group has shown through a *rns2‐2* null mutant that more total RNA is present in the cell comparison to WT, RNA accumulates in the vacuoles, and rRNA has an extended half‐life (Hillwig et al., 2011; Morriss et al., 2017). Both double null mutants *atg5‐1 rns2‐2* and *atg9‐4 rns2‐2* eliminate constitutive autophagy found in *rns2‐2*, but only *atg5‐1 rns2‐2* arrests vacuolar rRNA transport suggesting the importance of autophagy, especially ATG5, in selective ribosome turnover (Floyd et al., 2015). Dr. MacIntosh's group subsequently found, through use of a fluorescent RNA assay and a null mutant of RNA exosome‐associated DExD/H box RNA helicase *SUPERKILLER2* (AtSKI2), that *ski2‐5* null mutant plants have reduced ability to transport fluorescent RNA to vacuoles (Floyd et al., 2022). This transport is ATP dependent which mimics the RNautophagy mechanism in mammals. The summation of these works indicates three distinct rRNA turnover pathways—macroautophagy via RNS2, selective ribosomal autophagy via ATG5, and a plant RNautophagy‐like mechanism via SKI2.

### 1.4 RNA control of gene expression

Eukaryotic organisms have evolved several mechanisms to alter the expression of genetic loci in developmental or stress‐specific manners through sRNA. The symposium featured talks on two major pathways of sRNA‐mediated control: sRNA silencing transposons through the deposition of chromatin modification marks from Dr. Julie Law (Salk Institute, USA) and Dr. Zofia Szweykowska‐Kulinska's (Adam Mickiewicz University, Poland) work understanding microRNA‐mediated response to abiotic stress in barley.

Global DNA methylation patterning has emerged as a critical layer of genetic regulation with the establishment, removal, and regulation of these marks indicative of a complex and highly regulated evolved mechanism in Arabidopsis and other eukaryotes (Bartels et al., 2018; Law & Jacobsen, 2010). Dr. Julie Law discussed these mechanisms in the context of the discovery and annotation of Arabidopsis chromatin remodeling factors, CLASSY (CLSY) 1–4, mechanistically placed by her group as acting with RNA polymerase‐IV (Pol IV) to control production of 24‐nucleotide small‐interfering RNAs through the RNA‐directed DNA methylation (RdDM) pathway (Zhou et al., 2018). Interestingly, she reported on locus‐specific behavior for individual CLSY proteins. In particular, she noted specific connections between individual CLSY proteins and chromatin marks, as well as specific expression patterns of individual CLSY proteins, demonstrating CLSYs potential as tissue‐specific regulators of DNA methylation (Zhou et al., 2022). Analysis of specific CLSY knockout mutants demonstrated that CLSYs act as locus‐specific coregulators of the RdDM pathway in diverse tissues. There is a continuing focus on characterizing loci specific motifs associated with CLSY specificity. In combination with previously reported CLSY‐specific epigenomic associations, a model is emerging where both genetic (sequence‐specific motifs) and epigenetic (GG/H3K9) information are essential components involved in target loci regulation and tissue‐specific epigenetic patterning.

Dr. Zofia Szweykowska‐Kulinska discussed a different class of small ncRNAs, 21‐nt microRNAs (miRNAs), as a major class of gene expression regulators in the context of drought stress in barley. Her group's tissue‐specific identification of novel miRNAs and their targets through sRNA‐seq and degradome seq target suggest an important involvement of microRNAs in the development and function of floral organs as well as abiotic stress (Smoczynska et al., 2020). Six novel barley microRNAs were detailed, and their differential expression during induced drought implicates them and their targets as critical components of barely's response to drought stress. Of interest was microRNA hvu‐x13 targeting a TRP domain‐containing protein, this domain implicated in drought tolerance in Arabidopsis (Rosado et al., 2006). Additionally, microRNA hvu‐x8 targets a SWIRM domain‐containing protein, a domain also found in the canonical SWI/SNF‐family of chromatin remodelers, with predicted implication involved in chromatin–protein complexes critical during drought tolerance.

### 1.5 Plastid transcription

A plastid is an endosymbiotic organelle with its own genome. The plastid genome retains structural and organizational features from its prokaryotic ancestors but has also acquired unique features over a billion years of coevolution with the nucleus. One of the features of plastid DNA is that it congregates into chromatin‐like structures termed nucleoids. Dr. Andrzej T. Wierzbicki (University of Michigan, USA) tackled how plastid DNA is structurally organized (Palomar et al., 2022). Dr. Wierzbicki's team discovered that actively transcribed DNA in the plastid is membrane bound. This observation led to the proposal of a layered structure of plastid nucleoids, composed of an active membrane‐associated core and less transcriptionally active peripheral region. The team explored what determines how close DNA attaches to the membrane. Plastid‐encoded RNA polymerase (PEP) is essential for plastid gene transcription, during which PEP is recruited to the gene promoter. Nuclear‐encoded sigma factors (SIGs) are required for recruitment of PEP to genes' promoters. Dr. Wierzbicki's team disclosed that disruption of *SIG2* and *SIG6* causes certain gene sets to reduce their association with the membrane, leading to disruption of their transcription. This indicates that RNA polymerase activity promotes DNA tethering to the membranes. Put together, Dr. Wierzbicki's team uncovered a sigma factor‐mediated, gene‐specific activity in organizing plant nucleoids via affecting membrane association.

### 1.6 RNA and translation

Upstream ORFs (uORFs) are open reading frames that have start codons contained within the 5′ untranslated region (Zhang et al., 2021). In animals, uORFs are known to reduce mRNA stability through the nonsense‐mediated decay (NMD) pathway and to compete against their respective ORFs during translation (Zhang et al., 2019). Dr. Polly Hsu (Michigan State University, USA) reported that translated uORFs (TuORFs) in Arabidopsis and tomato express higher levels of transcripts than its main ORF but, even more intriguingly, show a 38% reduction in translation efficiency, a 14% reduction in mRNA half‐lives, and 43% reduction in protein levels of the main ORF. The observed transcriptional increase in plant TuORFs was consistent throughout different developmental stages and tissue types. Moreover, TuORF mRNAs have expression patterns distinct from NMD targets and do not show increased transcript levels in NMD mutants unlike NMD targets—which suggests these plant TuORFs are processed by a decay pathway distinct from NMD in comparison with their animal counterparts.

Dr. Sjors van der Horst (University of California, Riverside, USA) presented his research on conserved peptide uORFs (CPuORFs), specifically bZIP11's CPuORF function in ribosome stalling in high sucrose concentration leading to lower translation for bZIP11's main ORF (van der Horst et al., 2020). Mutational analysis, altering single amino acids in the CPuORF's C‐terminal domain, showed bZIP11's CPuORF is required for ribosome stalling in vitro while cryo‐electron microscopy showed sucrose binding within the ribosome exit tunnel which allows the nascent peptide of bZIP11's CPuORF to interact with its ribosomal binding partners. Based on cryo‐EM, no observable release factor could be found bound to the ribosome. The evidence, taken together, suggests bZIP11's CPuORF can inhibit translation termination in a metabolite‐dependent manner.

### 1.7 Short and long ncRNAs

High‐throughput sequencing has revealed 80% to 90% of plant transcripts are ncRNAs (Kapranov et al., 2007). ncRNAs are RNA molecules that do not encode a protein. They are further divided into two classes based on size. Small ncRNA is shorter than 200 nucleotides, such as short‐interfering RNAs (siRNAs), which are commonly 21–24 nucleotides long, as well as other classes including microRNAs. ncRNAs with more than 200 nucleotides in length are defined as long ncRNAs (lncRNAs). Despite the weak coding potential of these molecules, the basis of eukaryotic complexity and phenotypic variation may lie in ncRNAs, or the crosstalk between them, at least in part executed via chromatin remodeling. Thomas Roulé (Université Paris‐Saclay, France) revealed a lncRNA, and marneral silencing (MARS) modulates the epigenetic reprogramming of the marneral cluster in response to abscisic acid (Roulé et al., 2022). Functionally related genes for secondary metabolites biosynthesis form clusters. Several metabolic clusters have been identified in Arabidopsis, including that of marneral, a secondary metabolite crucial for plant development. Within the marneral cluster, Dr. Roulé's team identified MARS, a lncRNA involved in the control of the gene expression in this cluster. In response to ABA, MARS locally modified the epigenetic status, including H3K27me3 deposition and chromatin condensation, of the cluster. In addition, MARS promotes the formation of a chromatin loop joining the first gene of the biosynthetic pathway and a distal ABA‐responsive enhancer. These findings demonstrate that lncRNAs add a new regulatory layer for metabolic gene clusters, and it may drive the evolution of biosynthetic pathways.

Short ncRNAs are also being discovered to regulate gene expression at the epigenetic level. Rebecca Mosher (University of Arizona, USA) discussed that abundant 24‐nt siRNAs are produced from a small number of “siren” loci in ovules (Burgess et al., 2022; Grover et al., 2020). Fewer than 200 siren loci account for over 90% of siRNAs in ovules and early seeds, and these siRNAs primarily arise from gene fragments embedded in these loci. Dr. Mosher's team showed that these siren siRNAs trigger DNA methylation at homologous protein‐coding genes despite the presence of mismatches between the siRNA and the target locus. In some cases, this *trans*‐methylation impacts expression of the target protein‐coding gene. In the endosperm, siren siRNAs are maternally biased. They speculate that these siRNAs might be moving from the maternal diploid seed coat into the developing endosperm to direct DNA methylation, thus influencing gene expression in filial tissues. These observations suggest a potential mechanism for sporophytic control over next generation, via epigenetic regulation from maternally derived siren siRNAs.

Regulation of chromatin modifications and gene expression is also conducted in part by other sRNAs such as 24‐nt small‐interfering RNAs (siRNAs). Dr. Mary Gehring (Whitehead Institute for Biomedical Research) reported the effects of parental Pol IV activity and the resulting developmental impacts on seed fitness and gene expression within the endosperm of Arabidopsis. Pol IV functions in the RdDM pathway by producing short, noncoding transcripts that are eventually processed into 24‐nt sRNAs. Dr. Gehring's work focused on NRPD1, a subunit of Pol IV, which previous studies have shown to play a major role in endosperm gene expression. Using a series of homozygous and heterozygous *nrpd1* crosses, her research demonstrated that the expression of maternal or paternal Pol IV has clear impacts on endosperm, with some expression patterns exhibiting antagonistic tendencies (Satyaki & Gehring, 2022).

### 1.8 Prokaryotic RNA tools and interactions

Since the initial discovery of the CRISPR‐Cas9 system, significant advances have been made in manipulating this system for the purposes of genetic engineering. The Cas9 nuclease will bind a single‐guide RNA (sgR) that it uses to scan the genome for the complement in the organism in which it is expressed (Chen et al., 2019). At its target site it will make a nick where mutations, deletions, or new sequences can be introduced (Chen et al., 2019). While base editing and gene activation have been made possible via modifications to this method, not until recently have systems been created that allows for simultaneous occurrence of both. (Chen et al., 2019; Molla et al., 2021; Pan et al., 2021). Dr. Yiping Qi (University of Maryland, USA) discussed a CRISPR‐combo system his lab developed to allow for concurrent base editing and gene activation in plants. By altering the length of the protospacer in the sgR to either 15 (short) or 20 (long) nucleotides, they were able to control the activity of the system to dictate whether editing or activation occurred: The short spacer allowed only gene activation, while the long spacer produced successful editing (Pan et al., 2022). By expressing both guide RNAs with an active Cas9‐deaminase fusion protein and MS2‐SunTag‐activator, they were able to edit and activate two different genes in tomatoes and rice protoplasts selectively and simultaneously (Pan et al., 2022). Following their successful trial experiments, they moved on to accelerating flowering in Arabidopsis and regeneration in both rice and poplar (Pan et al., 2022). The future aspiration is to utilize this method to speed up crop testing so that new cultivars can be brought to market sooner.

### 1.9 RNA modifications

The collection of RNA modifications, or covalent additions of chemical moieties to RNA, far exceeds the number of known DNA modifications and forms the epitranscriptome which regulates RNA metabolism, affecting several properties such as RNA secondary structure, translation rate, and RNA stability (Roundtree et al., 2017; Yu, Sharma, et al., 2021). One internal modification, N6‐methyladenosine (m6A), has garnered significant attention as the most abundant mRNA modification equipped and dynamically regulated by a set of proteins known as writers (methyltransferases) that apply m6A, erasers (demethylases) that remove m6A, and readers that are proteins that recognize m6A and signal downstream targets (Meyer & Jaffrey, 2017). m6A plays a crucial role in both animal and plant development (Hongay & Orr‐Weaver, 2011; Shen et al., 2016).

Dr. Chuan He (University of Chicago and HHMI, USA) reported that the fat mass and obesity‐associated protein FTO, an RNA methylase, is responsible for demethylating m6A long‐interspersed element‐1 (LINE1) ultimately leading to chromatin state alterations and changes in gene expression that affect mouse oocyte/embryonic development (Wei et al., 2022). Dr. He also described the development of transgenic rice and potato lines expressing FTO (Yu, Liu, et al., 2021). In field trials, these FTO‐expressing transgenic rice and potato lines showed an augmented yield and biomass by approximately 50% compared with non‐transgenics. Dr. Brian Gregory (University of Pennsylvania, USA) reported that salt stress‐induced m6A deposition stabilizes transcripts that are then less prone to ribonucleolytic cleavage in Arabidopsis (Anderson et al., 2018). Moreover, salt stress‐induced m6A deposition decreases the transcript's secondary structure ultimately contributing to higher protein levels (Kramer et al., 2020).

### 1.10 RNA structure

The function of an RNA can be intimately linked with its structure. While DNA predictably forms a double stranded helix, RNA can adopt numerous conformations allowing it to serve many roles (Bevilacqua et al., 2016). The structure of an RNA can be impacted by many environmental factors such as temperature, pH, and salt concentrations which plants experience as abiotic stressors. Dr. Philip Bevilacqua (Pennsylvania State University, USA) and Dr. Sarah Assmann (Pennsylvania State University, USA) both discussed the effects abiotic stressors have on the RNA structurome, defined here as the structures of all expressed RNAs, in plants.

Comparing the folding change in transcripts from Arabidopsis exposed to 100 mM NaCl to simulate increased soil salinity with those from plants grown in the absence of any stress, Dr. Bevilacqua shared an interesting correlation they observed between concordancy and abundance (Tack et al., 2020). Concordancy occurs in an RNA when it experiences uniform changes in structure across both the coding and untranslated regions under stress (Tack et al., 2020). When concordant exposure in Arabidopsis was noted, the RNA became more single stranded and tended to decrease in abundance when experiencing salt stress. This was attributed to the transcript becoming more accessible to the degradation machinery as it unfolded. Conversely, those RNAs that demonstrated concordant protection had a higher degree of structure under stress, were better able to resist degradation, and so increased in abundance. GO analysis revealed these transcripts were enriched in salt stress response genes. What is most striking is that Dr. Assmann, who is a co‐author with Dr. Bevilacqua, the same pattern from studies they conducted with heat shocked rice, suggesting that this may be a global response shared between RNAs of plants if not more widely amongst eukaryotes (Su et al., 2018).

Dr. Assmann also presented work highlighting the relationship between structure‐altering single‐nucleotide polymorphisms (SNPs) (riboSNitches) and climatic factors in Arabidopsis. The *ZINC RIBBON 3* (*ZR3*) transcript is an example of a riboSNitch possessing a G to A SNP that alters its secondary structure (Ferrero‐Serrano et al., 2022). It was found that there was a correlation between the frequency of this SNP for *ZR3* and the plants' geographical location away from the coast; plants further inland that experienced a wider range of fluctuating temperatures contained a higher percentage of the G to A variant than those growing near the coast. They then broadened the scope of their study to predict riboSNitches across the Eurasian Arabidopsis accessions and search for correlations with various climatic factors, with the findings summarized in their searchable CLIMtools app. In the future they are planning to expand the CLIMtools database to contain other plant species and weather‐related events.

### 1.11 *Trans*‐species RNA interactions

Presentations on the role of RNA in plant interactions with other organisms were focused on sRNAs generated in one organism and functioning in another. This phenomenon has been studied in the context of sRNAs traveling between interacting organisms with the outcome of inducing transgene silencing of pathogen and parasite mRNAs and suppressing host immune response (Baulcombe, 2004; Huang et al., 2019). Dr. Michael Axtell (Pennsylvania State University, USA) discussed *trans*‐species miRNAs in a plant–plant interaction, and Dr. Hailing Jin (University of California, Riverside, USA) presented findings detailing the mobility and function of sRNAs traveling between organisms of differing taxonomic kingdoms in a plant–pathogen model.

Dr. Michael Axtell detailed work with the parasitic plant *Cuscuta campestris* as a model to study plant–plant RNAi (Johnson et al., 2019; Shahid et al., 2018). They reported finding 76 *Cuscuta* miRNAs significantly upregulated in the interface region of *Cuscuta* and host Arabidopsis, further refining this set using complementary host small‐RNA‐seq to reveal six Arabidopsis mRNAs containing plausible miRNA‐complementary sites with the significantly upregulated siRNAs in the host–parasite interface. Utilizing host plants deficient in DCL4‐ (DICER‐LIKE 4) and RDR6‐ (RNA‐DEPENDENT RNA POLYMERASE/SGS2), they measured differential abundance of each siRNA to confirm that parasite *Cuscuta*‐derived miRNA activity is dependent on host machinery. Target mRNAs included *TIR1* (*TRANSPORT INHIBITOR RESPONSE 1*) and *AFB2* (*AUXIN SIGNALING F‐BOX 2*) involved in bacterial pathogenesis and defense signaling (Robert‐Seilaniantz et al., 2011) and *SEOR1* (*SIEVE ELEMENT OCCLUSION AMINO‐TERMINUS PROTEIN*) involved in reducing sap loss after wounding (Knoblauch et al., 2014). Additional work revealed conservation of *trans*‐species sRNAs in multiple *Cuscuta* species with predicted targets in several hosts (Johnson et al., 2019). Interestingly nearly all the *Cuscata trans*‐species miRNAs target regions within host mRNA containing high levels of conservation, implying a robust approach for a parasite to maintain a broader host range. Further work defining the timing of *Cuscuta*‐host sRNA transfer, with specific interest on the adhesive phase of parasite binding initiating the window to sRNA conduction is ongoing.

Dr. Hailing Jin presented an elegant series of experiments detailing the mechanism of plant‐mediated sRNA delivery via extracellular vesicles (EVs) (Cai et al., 2018; He et al., 2021) in the pathogen–host Arabidopsis–*Botrytis cinerea* system. An innovative protocol to isolate pure fungal cells from infected tissue, coupled with plant‐EV isolation, enabled Cai et al., 2018 to test plant–pathogen sRNA transfer. Pure fungal cell isolation allowed for the detection and profiling of Arabidopsis sRNAs in *B. cinerea*, corroborated with the purification and profiling of Arabidopsis EVs, revealed a 73.8% overlap between the two detectable sRNA populations. *Dcl2/3/4* and *rdr6‐15* mutant experimentation revealed the significance of *trans*‐acting siRNAs, both mutants demonstrating increased susceptibility to *B. cinerea*. More recent work from Dr. Jin identified key RNA‐binding proteins in plant EVs, showing AGO1, RH11, and RH37 interaction with EV‐enriched sRNAs noted above (He et al., 2021). AGO1 specifically was shown to bind to 20–22 nucleotide sRNAs containing 5′‐terminal Us in Arabidopsis EVs, suggesting AGO1 is in part responsible for sRNA specificity in the detected EVs. Continued work focuses on understanding the functional mechanism and destiny of these transferred sRNAs as well as understanding the protein and RNA signatures of the heterogenous population of plant RNA in secreted EVs.

## 2 CONCLUDING REMARKS

Contributions from this conference highlight the importance and necessity to further understand RNA properties due to RNA's expansive and diverse impacts within the cell, ability for communication outside the cell and between organisms, and potential for crop improvement. Novel RNA discoveries continue to deepen our understanding of both established and newly discovered pathways and processes. RNA phenomena in plants provide a unique perspective as parallel pathways in animals can differ to varying degrees, as evidenced by the research presented in this report. In turn, the continued development of tools such as CRISPR as illustrated by Dr. Yiping Qi's dual base editing and gene activation system, and RNA‐based crop improvement methods, shown by Dr. Zofia Szweykowska‐Kulinska's work in barley miRNA and Dr. Chuan He′s work in potato and rice through RNA modifications, can be used to address current biological and agricultural problems. The field of RNA biology, especially in plants, remains a research area full of potential discoveries to improve crop systems, reveal novel aspects of established pathways, and identify noncanonical processes.

## CONFLICT OF INTEREST

The authors declare no conflict of interest associated with the work described in this manuscript.
